# Supplementary material for: Preparation of Nitrogen and Sulfur Co-Doped Fluorescent Carbon Dots from Cellulose Nanocrystals as a Sensor for the Detection of Rutin
Source: Molecules. 2022 Nov 18;27(22):8021. doi: 10.3390/molecules27228021 (PMC9697528; doi:10.3390/molecules27228021)
Supplement: Supplementary file 1 [file molecules-27-08021-s001.zip › molecules-1998914-supplementary.pdf]

Preparation of Nitrogen and Sulfur Co-Doped Fluorescent Carbon Dots from Cellulose Nanocrystals as a Sensor for the Detection of Rutin

Supplementary Materials

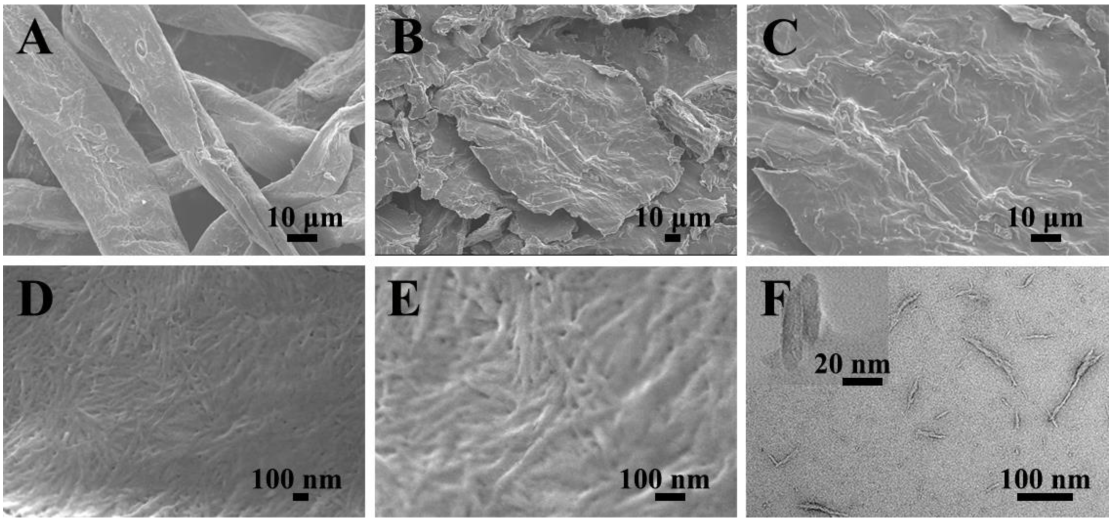

Figure S1. SEM images of cellulose (A, B, C) and CNC (D, E), TEM images of CNC (F)

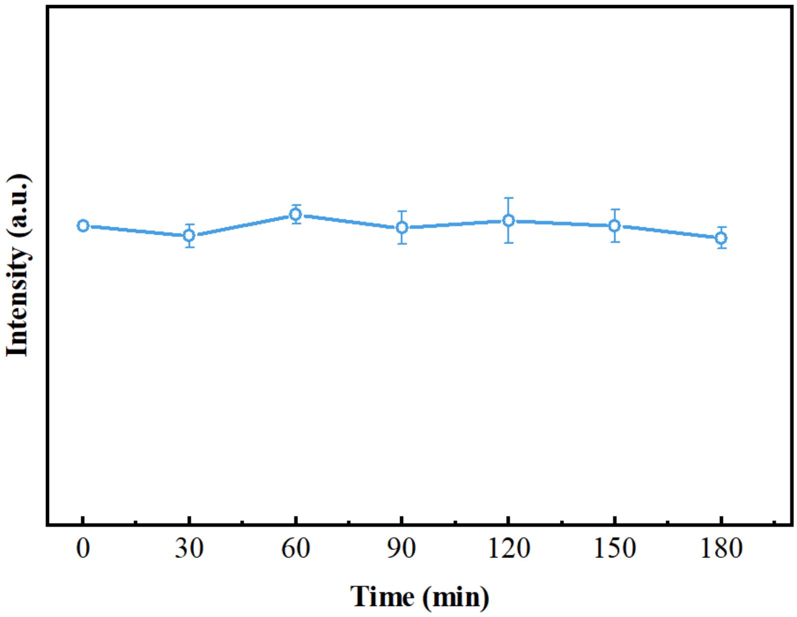

Figure S2. the effect of UV irradiation time on the fluorescence intensity of N, S-CDs

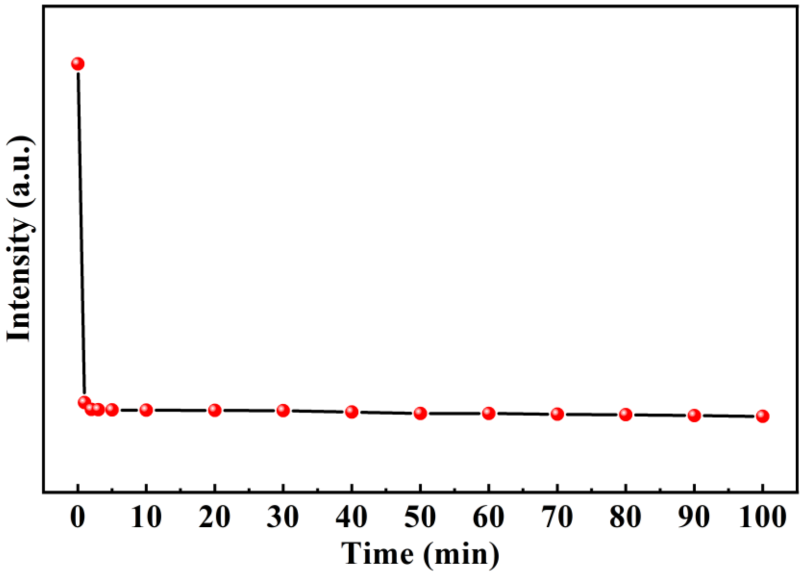

Figure S3. Time-dependent fluorescence changes of N, S-CDs in the presence of 45 mg/mL rutin.

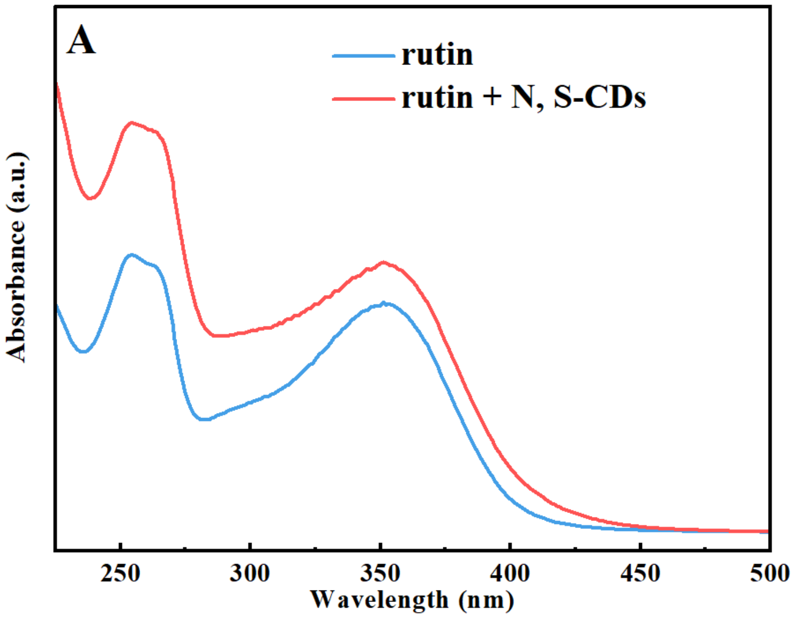

Figure S4. UV-Vis spectra of rutin in the presence and absence of the N, S-CDs.

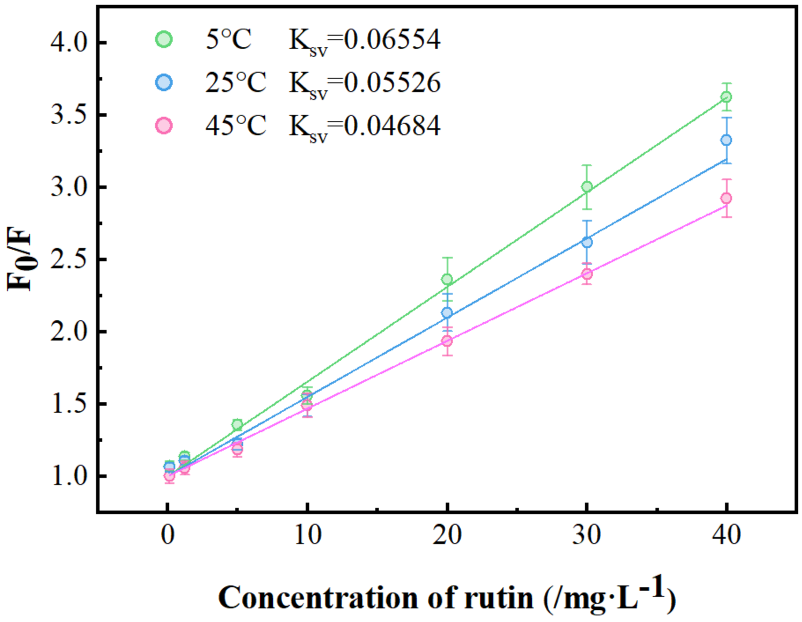

Figure S5. Stern-Volmer plots at different temperatures
